# Parental Vaccine Hesitancy in the Post-COVID-19 Era: Results from a Cross-Sectional Survey in the United States, 2025

**DOI:** 10.3390/vaccines14070601

**Published:** 2026-07-08

**Authors:** Syeda Sharmin Duza, Christine Crudo Blackburn, Mayra Rico, Matthew R. Boyce

**Affiliations:** 1 USA Center for Rural Public Health Preparedness, Texas A&M University School of Public Health, College Station, TX 77843, USA; 2 Department of Health Policy & Management, Texas A&M University School of Public Health, College Station, TX 77843, USA

**Keywords:** child health, communicable disease, immunization, vaccine, vaccine confidence

## Abstract

**Background/Objectives:** Vaccine hesitancy has long been recognized to pose a significant barrier to achieving optimal immunization coverage and as a leading public health threat. However, much of the existing research on parental hesitancy toward routine childhood vaccines was conducted prior to the COVID-19 pandemic. Acknowledging the impact that this public health emergency had on attitudes toward vaccination, there is a need to conduct additional research into contemporary predictors of parental vaccine hesitancy. **Methods:** We conducted a survey in August 2025 to characterize vaccine hesitancy attitudes in parents in the United States. A series of 10 statements about vaccines was presented to study participants, who were asked to indicate their agreement with the statements using 5-point Likert scales. A vaccine hesitancy index measure was then calculated by averaging the scores, where higher scores corresponded to higher levels of vaccine hesitancy. A hierarchical regression analysis was then conducted to identify participant characteristics that were associated with vaccine hesitancy. **Results:** A total of 1016 individuals were included in the analysis. Simple linear regression suggested that education, annual household income, and politics were associated with vaccine hesitancy. The fully adjusted multiple linear regression model that accounted for participant demographic, socioeconomic, geographic, and political characteristics suggested significant associations between race, education, and partisanship. **Conclusions:** Significant associations exist between parental vaccine hesitancy and race, education, and partisanship. These results contribute to our understanding of vaccine hesitancy in a post-COVID-19 world and suggest that social, educational, and political factors meaningfully influence parental vaccine hesitancy in the United States.

## 1. Introduction

Vaccines are widely considered to be among the greatest achievements in public health [1]. Vaccination programs have contributed to the substantial decline in morbidity and mortality from once common infectious diseases—such as diphtheria, measles, mumps, and polio—and are also responsible for the eradication of smallpox. However, despite the extensive benefits of vaccination, many parents harbor concerns about the safety and efficacy of childhood vaccines [2,3,4,5,6,7]. Many of these concerns are fueled by discredited research suggesting associations between vaccinations and autism [8,9]. Still, these concerns can result in a hesitancy toward childhood vaccines characterized by uncertainty in the acceptance of vaccines, delaying the acceptance of vaccines, only accepting certain vaccines, or refusing vaccines altogether, despite the availability of vaccination services [10,11].

Vaccine hesitancy has long been recognized as a leading threat to global health due to its potential to reduce vaccine uptake and undermine disease prevention efforts [6,12,13,14]. However, the COVID-19 pandemic fundamentally altered the context surrounding vaccine decision-making, and by extension, vaccine hesitancy. In the United States, decisions surrounding the COVID-19 vaccine were markedly divided across demographic, socioeconomic, political, and geographic lines [15,16]. For example, evidence suggests that COVID-19 vaccine attitudes were strongly shaped by educational attainment, partisanship, trust in healthcare systems, perceived risk, and exposure to health misinformation [15]. Previous research has also found higher levels of COVID-19 vaccine hesitancy and lower vaccination coverage among rural populations when compared to urban populations [16]. Critically, work has also demonstrated that there is spillover between beliefs about COVID-19 vaccines and routine childhood vaccines [17,18].

Despite the substantial amount of research that has already been conducted on vaccine hesitancy, there remains a need for additional research that examines demographic, socioeconomic, geographic, and political predictors of vaccine hesitancy attitudes in the aftermath of the COVID-19 pandemic. Understanding the relative contribution of these factors is essential for developing targeted, evidence-based, and relevant public health interventions and communication strategies. To this end, this study examines demographic, socioeconomic, geographic, and political factors that are associated with parental vaccine hesitancy attitudes in the United States. Recognizing the current context surrounding vaccines and decisions to vaccinate, we hypothesize that race, education, partisanship, and rurality will be associated with parental vaccine hesitancy.

## 2. Data & Methods

This study used a cross-sectional survey design to identify factors associated with parental vaccine hesitancy attitudes in the United States. Eligibility criteria for participation in the study included: (1) being 18 years of age or older, (2) residing in the United States, and (3) being the parent or legal guardian of at least one child 5 years of age or younger.

### 2.1. Survey Questionnaire & Variables

We created a survey questionnaire that used a validated survey tool to characterize vaccine hesitancy attitudes [19]. A series of 10 statements about vaccines was presented, and study participants were asked to indicate their agreement using 5-point Likert scales (i.e., (1) Strongly agree, (2) Agree, (3) Neither agree, nor disagree, (4) Disagree, (5) Strongly disagree). The scales were designed so that higher scores corresponded to higher levels of vaccine hesitancy attitudes (e.g., strongly agreeing that children do not need vaccines for diseases that are not common anymore). Three of these statements were presented in a fashion that necessitated a reverse coding scheme. These included: “New childhood vaccines carry more risks than older vaccines,” “I am concerned about serious, long-term adverse effects of childhood vaccines,” and “My child/children do not need vaccines for diseases that are not common anymore.” A Cronbach’s alpha coefficient was calculated to determine the internal validity of these measures for the vaccine hesitancy scale, which was found to have a high level of consistency (α = 0.86). The arithmetic mean of responses to these questions was calculated as a vaccine hesitancy index score, which was used as the dependent variable for data analyses.

The survey questionnaire also included questions relating to study participant characteristics, to be used as independent variables in data analyses. These variables included participant sex (male or female), age, race (i.e., American Indian or Alaska Native, Asian or Asian American, Black or African American, Native Hawaiian or Pacific Islander, White, or other), ethnicity (Hispanic or non-Hispanic), education (i.e., high school diploma or less, post-high school vocational training, some college but no degree, associate’s degree, bachelor’s degree, or graduate degree), annual household income ($49,999 or less, $50,000–$99,999, $100,000–$149,999, $150,000–$199,999, $200,000 to $249,999, $250,000 to $299,999, $300,000 to $349,999, $350,000 to $399,999, or $400,000 or more), health insurance status (insured or uninsured), partisan identity (strong Democrat, weak Democrat, lean Democrat, Independent, lean Republican, weak Republican, or strong Republican), and county and state of residence.

Race data were further condensed by combining American Indian or Alaska Native, Hawaiian or Pacific Islander, and other into a single category (i.e., other). Education data were condensed by combining post-high school vocational training and associate’s degree into a single category (i.e., technical certificate or associate’s degree). Annual household income data were condensed by combining several groups—$200,000 to $249,999, $250,000 to $299,999, $300,000 to $349,999, $350,000 to $399,999, and $400,000 or more—into a single category (i.e., $200,000 or more). Partisan identity data were condensed into a categorical variable by combining strong and weak Democrats into a single category (i.e., Democrat), Independents and lean Democrat/Republican into a single category (i.e., Independent), and strong and weak Republicans into a single category (i.e., Republican).

Participant residence data were used to categorize responses according to geographic region and metropolitan status. Standard definitions from the United States Census Bureau were used for categorizing geographic regions [20]. Participants’ county of residence was combined with 2023 rural–urban continuum codes (RUCCs) from the United States Department of Agriculture to categorize metropolitan status [21]. RUCCs categorize all US counties into 9 groups ranging from 1 (most urban) to 9 (most rural) based on urbanization, population size, and adjacency to metro areas. For this analysis, these data were further condensed by combining counties with RUCCs less than or equal to 3 into a single group (i.e., metropolitan) and RUCCs greater than or equal to 4 into a single group (i.e., non-metropolitan). The survey included several attention checks to promote data quality, and survey questions are available for review in the Supplementary Material (Supplementary File S1).

### 2.2. Sample Recruitment

Cint Marketplace was used to recruit participants matching the eligibility criteria for the study. Cint sources participants for online survey research using a double-opt-in procedure in which individuals opt into the Cint survey marketplace platform and then into specific surveys. Cint incentivizes participation in surveys using a points-based system that awards points for completing surveys; awarded points can later be redeemed for rewards. The surveys were administered in English using QualtricsXM from 20 August to 9 September 2025.

### 2.3. Study Population

The survey was initiated by 1590 individuals, but several responses were omitted for a variety of reasons. These omissions included 67 individuals (4.2%) who were excluded from data analyses for failing to consent to participate, and 228 individuals (14.3%) who were excluded due to data quality concerns (e.g., failing the attention check). Additionally, 279 (17.5%) individuals were excluded from data analyses due to missing data. Dropped responses never exceeded 10% of the total population for a single variable, avoiding the need to use imputation methods to address missing data. As a result, 1016 individuals comprised the study population. Study participants most often reported being female (n = 528, 52.0%), 25–34 years of age (n = 393, 38.7%), white (n = 648, 63.8%), non-Hispanic (n = 844, 83.1%), having attained a high school diploma or less (n = 300, 29.5%), an annual income of $50,000-$99,999 (n = 341, 33.6%), full-time employment (n = 688, 67.9%), maintaining health insurance (n = 976, 96.1%), a Democrat partisan identity (n = 458, 45.1%), and residence in the South (n = 397, 39.1%) (Table 1).

### 2.4. Data Analysis

Data analyses were conducted using StataBE v19, and the predetermined threshold for statistical significance was set at an alpha value of 0.05 (*p* < 0.05). Descriptive statistics were conducted to characterize vaccine hesitancy attitudes in the overall study population and subpopulations. Linear regression models were used to explore associations between vaccine hesitancy index scores and participant characteristics. Simple linear regression models with robust standard errors were used to investigate the association between vaccine hesitancy index scores and characteristics, where *y* represents the vaccine hesitancy index score, *β*_0_ represents the y-intercept, *β*_1_ represents the regression coefficient of the characteristic (*X*_1_), and ε represents the residual model error term (Equation (1)).

*y* = *β*_0_ + *β*_1_*X*_1_ + ε (1)

A hierarchical regression analysis was then conducted by progressively adding blocks of related independent predictor variables to linear regression models. Multiple linear regression models with robust standard errors were used to investigate the association between vaccine hesitancy index scores and characteristics, where *y* represents the vaccine hesitancy index score, *β*_0_ represents the y-intercept, *β*_1_–*β*_n_ represent the regression coefficients of independent variables (*X*_1_–*X*_n_) added to a given regression model, and ε represents the residual model error term (Equation (2)).

*y* = *β*_0_ + *β*_1_*X*_1_ + *β*_2_*X*_2_ + … *β_n_X_n_* + ε (2)

The first model added demographic variables and included participant sex, age, race, ethnicity, and education as independent variables. The second model added a block of socioeconomic variables and included participant sex, age, race, ethnicity, education, annual household income, and health insurance status as independent variables. The third model controlled for rural geography and included participant sex, age, race, ethnicity, education, annual household income, health insurance status, and rurality as independent variables. The fourth and final model included a block of political variables and included participant sex, age, race, ethnicity, education, annual household income, health insurance status, rurality, and partisanship as independent variables. In these models, sex, race, ethnicity, health insurance status, rurality, and partisanship were treated as categorical variables, while age, educational attainment, and annual household income were treated as continuous variables.

### 2.5. Ethical Review

Prior to initiating the recruitment of study participants, all study materials were submitted for ethical review. The study was declared exempt by the Texas A&M University Institutional Review Board (STUDY2025-0819). While this determination removed requirements to obtain informed consent, we sought consent from study participants as a best practice.

## 3. Results

The average vaccine hesitancy index score for the entire study population was 2.20 (SD = 0.71). Simple linear regression models suggested that participant sex, age, race, ethnicity, health insurance status, and rurality were not meaningfully associated with parental vaccine hesitancy attitudes (Table 2). Educational attainment, however, was found to be significantly associated with parental vaccine hesitancy attitudes, with higher levels of education associated with lower vaccine hesitancy attitudes (*β* = −0.055, 95% CI [−0.077, −0.033], *p* < 0.001). Higher annual household income was also found to be significantly associated with lower parental vaccine hesitancy attitudes (*β* = −0.047, 95% CI [−0.078, −0.016], *p* = 0.003). Lastly, partisanship was found to be meaningfully associated with parental vaccine hesitancy attitudes. Compared to Democrats (i.e., the reference group), study participants identifying as Independents (*β* = 0.286, 95% CI [0.153, 0.420], *p* < 0.001) and Republicans (*β* = 0.279, 95% CI [0.186, 0.372], *p* < 0.001) had significantly higher parental vaccine hesitancy attitudes.

In the first multiple linear regression model, which only included demographic variables, education was significantly associated with parental vaccine hesitancy attitudes (*β* = −0.056, 95% CI [−0.078, −0.033], *p* < 0.001), indicating that higher educational attainment was associated with lower vaccine hesitancy attitudes (Table 3). Sex, age, race, and ethnicity were not significantly associated with parental vaccine hesitancy attitudes.

The second model, which adjusted for socioeconomic variables, produced similar results. Significant associations between participant education and parental vaccine hesitancy attitudes remained (*β* = −0.051, 95% CI [−0.075, −0.026], *p* < 0.001). Sex, age, race, ethnicity, income, and health insurance status were not significantly associated with parental vaccine hesitancy attitudes.

The third multiple linear regression model also adjusted for rurality. In this model, education continued to demonstrate a significant, inverse association with vaccine hesitancy attitudes (*β* = −0.050, 95% CI [−0.075, −0.025], *p* < 0.001). Similar to the previous models, sex, age, race, ethnicity, income, and health insurance status were not significantly associated with parental vaccine hesitancy attitudes, nor was rurality.

The final, fully adjusted model included variables related to participant demographics, socioeconomic status, rurality, and politics. In this model, education remained significantly associated with lower parental vaccine hesitancy attitudes (*β* = −0.046, 95% CI [−0.071, −0.022], *p* < 0.001). Additionally, White participants demonstrated significantly lower vaccine hesitancy attitudes (*β* = −0.146, 95% CI [−0.243, −0.049], *p* = 0.003). Partisanship was also significantly associated with vaccine hesitancy attitudes in the fully adjusted model. Independent (*β* = 0.287, 95% CI [0.152, 0.421], *p* < 0.001) and Republican (*β* = 0.334, 95% CI [0.237, 0.433], *p* < 0.001) partisanship identities were associated with higher parental vaccine hesitancy attitudes when compared to Democrats. Associations between participant sex, age, ethnicity, income, insurance, and rurality were not statistically significant in the fully adjusted model.

## 4. Discussion

This study examined associations between parental characteristics and vaccine hesitancy attitudes in a large sample of parents in the United States. We hypothesized that vaccine hesitancy attitudes would be associated with multiple demographic, socioeconomic, geographic, and political factors—specifically, that race, educational attainment, partisanship, and rurality would be associated with parental vaccine hesitancy. Overall, our findings suggest that race, educational attainment, and partisanship were meaningfully associated with parental vaccine hesitancy attitudes, while rurality was not associated with vaccine hesitancy. A key contribution of this study is its examination of parental vaccine hesitancy in the post-COVID-19 era. Given the evolving environment surrounding vaccines and decisions to vaccinate since the COVID-19 pandemic, these findings provide updated evidence regarding the factors associated with vaccine hesitancy among parents in the United States.

Consistent with previous research on both hesitancy toward routine childhood immunizations [5,7] and COVID-19 vaccinations [15,22,23,24], we found a statistically significant association between race and vaccine hesitancy attitudes in the fully adjusted model, with white participants reporting lower vaccine hesitancy attitudes compared to non-white participants. Potential mechanisms underlying these observed associations may include racialized differences in trust toward healthcare systems, prior experiences with healthcare institutions, structural inequities in healthcare access, exposure to vaccine-related misinformation, and/or broader social and historical factors influencing trust in government and health authorities [13,25].

Previous research has yielded mixed results regarding the influence of education on vaccine hesitancy. In our study, parental educational attainment was consistently found to be inversely associated with vaccine hesitancy attitudes, with higher levels of education associated with lower vaccine hesitancy attitudes, even when adjusting for socioeconomic, geographic, and political factors. As suggested by others, this finding may be the result of individuals with higher educational attainment being more likely to accept vaccines and adhere to public health recommendations, possessing greater levels of health literacy, having improved understandings of scientific information, and maintaining higher levels of trust in healthcare systems [22,26,27]. However, previous research has also produced mixed findings, with some studies suggesting that education alone may not fully predict vaccine attitudes in highly polarized political or social environments [5,22]. Accordingly, this finding is notable because it suggests that the influence of education on vaccine behavior extends beyond simple socioeconomic advantage and may independently shape individuals’ ability to evaluate vaccine-related information and misinformation.

Furthermore, and as hypothesized, partisanship was also associated with parental vaccine hesitancy attitudes in both the simple and adjusted regression models. More directly, participants who identified as Independents or Republicans reported significantly higher vaccine hesitancy attitudes when compared to Democrats. These findings are aligned with the results of prior studies demonstrating increasing politicization of vaccine attitudes in the United States, particularly during and following the COVID-19 pandemic [28,29,30]. Several of these previous studies have suggested that political differences in vaccine attitudes may be partially explained by underlying demographic or socioeconomic factors, including education, geographic region, and media exposure. Still, our results suggest that political partisanship remains a significant predictor of parental vaccine hesitancy attitudes, even after adjustment for demographic, socioeconomic, and rurality-related variables. Mechanisms that underlie these associations may include partisan differences in media consumption patterns, trust in governmental and scientific institutions, ideological beliefs regarding individual autonomy and public health interventions, and differential exposure to vaccine-related misinformation within politically aligned information environments [31,32,33].

Contrary to our hypothesis and previous literature [16], we found no difference in vaccine hesitancy attitudes across rurality gradients. This is notable, as discourse has increasingly framed vaccine hesitancy as a phenomenon primarily impacting rural environments. In combination with the previously discussed results, what these findings suggest is that race, educational attainment, and partisanship may account for more variation in hesitancy attitudes than geographic rurality itself. Put differently, these findings suggest that vaccine hesitancy may be driven less by place of residence alone, and more by underlying social, educational, and political factors that shape trust, health literacy, and health-related decision-making.

The findings of this study have critical implications for public health practice and interventions focused on addressing vaccine hesitancy. Our results highlight the importance of developing targeted public health strategies that address underlying social, educational, and political influences on parental attitudes. Interventions aimed at improving vaccine confidence may benefit from emphasizing health literacy, culturally and politically appropriate communication strategies, and trust-building efforts within diverse communities. Additionally, the strong association between political partisanship and vaccine hesitancy underscores the need for public health messaging that is transparent, evidence-based, and capable of reaching individuals across the political aisle and increasingly polarized information environments. Collectively, these findings support the need for multifaceted approaches for promoting vaccination that account for the complex interaction between education, politics, trust, and other social determinants of health.

As with all research, there are several limitations that must be considered when interpreting these findings. First, the cross-sectional design limits our ability to establish causal relationships between participant characteristics and vaccine hesitancy attitudes. The observed associations should, therefore, be interpreted as correlational rather than causal. Second, participants were recruited through an online survey panel that utilized a double opt-in procedure. This recruitment strategy may have introduced selection bias, as it required study participants to opt in to the online panel, as well as this specific survey. As a result, these findings may hold limited generalizability to broader populations in the United States. The omission of participants who failed attention checks and provided incomplete surveys may also limit the external validity of these findings. Further, while efforts were made to obtain a diverse sample, certain demographic groups may have been over- or underrepresented relative to national population distributions. However, the absence of robust comparative benchmarks for parents with children under five years of age in the United States limits opportunities to compare our sample to population benchmarks and for survey weighting methods. Additionally, although the survey captured several important demographic, socioeconomic, geographic, and political variables, other potentially relevant factors such as previous vaccination behavior and child vaccination status, healthcare access, media consumption patterns, interpersonal trust, local public health messaging, religiosity, and exposure to misinformation were not directly assessed and may also influence vaccine hesitancy attitudes. Third, our study relied on self-reported survey responses. As such, responses may be subject to recall bias, response bias, and social desirability bias. Despite these limitations, our findings contribute to the growing literature on vaccine hesitancy and represent a consequential update to the literature in a post-COVID-19 world. The consistency of the associations between educational attainment and vaccine hesitancy across all regression models strengthens confidence in the robustness of the findings. Similarly, the persistence of political partisanship as a significant predictor after adjusting for demographic, socioeconomic, and geographic variables suggests that the observed relationships are unlikely to be entirely explained by confounding alone.

Future research should continue to explore the complex social, educational, and political factors that shape vaccine hesitancy, particularly within diverse demographic, socioeconomic, and geographic populations. Longitudinal and mixed-methods studies may also help clarify how vaccine attitudes evolve across time and provide deeper insights into the mechanisms through which factors such as education, partisanship, trust, and misinformation influence parental decisions surrounding childhood vaccines.

## 5. Conclusions

In this large sample of parents in the United States, race, educational attainment, and partisan identity were found to be meaningfully associated with parental vaccine hesitancy. And, contrary to *a priori* expectations, rurality was not significantly associated with vaccine hesitancy. As vaccine hesitancy continues to pose challenges for vaccination programs in the United States in the wake of the COVID-19 pandemic, these findings highlight the need for inclusive and evidence-based interventions focused on health literacy, trust-building, and public health communication to improve vaccine confidence and uptake.

## Figures and Tables

**Table 1:** Demographic, socioeconomic, and other characteristics of the study population (n = 1016).

Characteristic	n (%)
Sex	
Female	528 (52.0)
Male	401 (39.5)
Prefer not to answer	87 (8.6)
Age	
18–24 years	97 (9.5)
25–34 years	393 (38.7)
35–44 years	357 (35.1)
45–54 years	108 (10.6)
55–64 years	39 (3.8)
65 years or older	22 (2.2)
Race	
Asian or Asian American	96 (9.4)
Black or African American	166 (16.3)
Other	106 (10.4)
White	648 (63.8)
Hispanic ethnicity	
Hispanic	172 (16.9)
Non-Hispanic	844 (83.1)
Educational attainment	
High school or less	300 (29.5)
Some college, no degree	146 (14.4)
Associate’s degree or technical certificate	147 (14.5)
Bachelor’s degree	235 (23.1)
Graduate degree	188 (18.5)
Household income, US$	
$49,999 or less	337 (33.2)
$50,000-$99,999	341 (33.6)
$100,000-$149,999	172 (16.9)
$150,000-$199,999	94 (9.2)
$200,000 or more	72 (7.1)
Health insurance status	
Insured	976 (96.1)
Uninsured	40 (3.9)
Rurality	
Metropolitan	842 (82.9)
Non-metropolitan	174 (17.1)
Partisanship	
Democrat	458 (45.1)
Independent	138 (13.6)
Republican	420 (41.3)
Geographic region	
Midwest	215 (21.2)
Northeast	177 (17.4)
South	397 (39.1)
West	227 (22.3)

**Table 2:** Associations between respondent characteristics and parental vaccine hesitancy attitudes from simple linear regression models (n = 1016).

Characteristic	Coef. [95% CI]
Sex	0.002 [−0.092, 0.096]
Age	−0.002 [−0.055, 0.028]
White	−0.045 [−0.136, 0.046]
Hispanic ethnicity	0.073 [−0.045, 0.190]
Educational attainment	*** −0.055 [−0.077, −0.033]
Annual household income	** −0.047 [−0.078, −0.016]
Health Insurance	−0.136 [−0.362, 0.089]
Rurality	0.048 [−0.068, 0.165]
Independent partisan identity	*** 0.286 [0.153, 0.420]
Republican partisan identity	*** 0.279 [0.186, 0.372]

** *p* < 0.01, *** *p* < 0.001.

**Table 3:** Regression coefficients from multiple linear regression models examining associations respondent characteristics and parental vaccine hesitancy attitudes (n = 1016).

Characteristic	Model 1	Model 2	Model 3	Model 4
	D	D+SES	D+SES+R	R+D+SES+P
Sex	−0.014	−0.019	−0.020	−0.021
Age	−0.000	−0.000	−0.000	−0.000
White	−0.029	−0.031	−0.032	** −0.146
Hispanic ethnicity	0.078	0.076	0.077	0.078
Educational attainment	*** −0.056	*** −0.051	*** −0.050	*** −0.046
Annual household income		−0.015	−0.015	−0.018
Health Insurance		−0.055	−0.055	−0.062
Rurality			0.022	−0.014
Independent partisan identity				*** 0.287
Republican partisan identity				*** 0.335

D: Demographics, P: Politics, R: Rurality, SES: Socioeconomics, ** *p* < 0.01, *** *p* < 0.001.

## Data Availability

The data supporting the conclusions of this manuscript will be made available by the corresponding author upon reasonable request.

## Supplementary Materials

The following supporting information can be downloaded at: https://www.mdpi.com/article/10.3390/vaccines14070601/s1, File S1: Survey Questions.
