# Supplementary material for: Parental Vaccine Hesitancy in the Post-COVID-19 Era: Results from a Cross-Sectional Survey in the United States, 2025
Source: Vaccines (Basel). 2026 Jul 8;14(7):601. doi: 10.3390/vaccines14070601 (PMC13419274; doi:10.3390/vaccines14070601)
Supplement: Supplementary file 1 [file vaccines-14-00601-s001.zip › vaccines-4409937-supplementary.pdf]

Supplement File S1. Survey Questions.

If you wish to participate, please click the "I agree" button and you will be taken to the survey. If you do not wish to participate, please select "I disagree" or select the X in the corner of the window to close your browser.

o I agree
o I disagree

We care about the quality of our survey data. For us to get the most accurate measures of your opinion, it is important that you provide thoughtful answers to the questions in this survey. Do you commit to providing thoughtful answers to the questions in this survey?

o I cannot promise either way
o Yes, I commit to providing thoughtful answers
o No, I will not commit to providing thoughtful answers

Vaccine Hesitancy Question Block
Please indicate how much do you agree with the each of the following statements about childhood vaccinations.

Q1. Childhood vaccines are important for my child/children's health.

o Strongly disagree
o Disagree
o Neither agree nor disagree
o Agree
o Strongly agree

Q2. Childhood vaccines are effective.

o Strongly disagree
o Disagree
o Neither agree nor disagree
o Agree
o Strongly agree

Q3. Having my child/children vaccinated is important for the health of others in my community.

o Strongly disagree
o Disagree
o Neither agree nor disagree
o Agree
o Strongly agree

Q4. All childhood vaccines offered by my child’s health care provider are beneficial.

o Strongly disagree
o Disagree
o Neither agree nor disagree
o Agree
o Strongly agree

Q5. New childhood vaccines carry more risks than older vaccines.
o Strongly disagree
o Disagree
o Neither agree nor disagree
o Agree
o Strongly agree

Q6. The information I receive about childhood vaccines from their health care providers is reliable and trustworthy.
o Strongly disagree
o Disagree
o Neither agree nor disagree
o Agree
o Strongly agree

Q7. Getting vaccines is a good way to protect my child/children from disease.
o Strongly disagree
o Disagree
o Neither agree nor disagree
o Agree
o Strongly agree

Q8. I generally do what my doctor or health care provider recommends about vaccines for my child/children.
o Strongly disagree
o Disagree
o Neither agree nor disagree
o Agree
o Strongly agree

Q9. I am concerned about serious, long-term adverse effects of childhood vaccines.
o Strongly disagree
o Disagree
o Neither agree nor disagree
o Agree
o Strongly agree

Q10. My child/children do not need vaccines for diseases that are not common anymore.
o Strongly disagree
o Disagree
o Neither agree nor disagree
o Agree
o Strongly agree

Q11. Sometimes surveys can be dull, and attention can wander. To let us know that you are still paying attention, please select fruit from the list of foods below.
o Egg

o Blueberry
o Pizza
o Ice cream
o Fish

Demographic Question Block

Q12. Where do you currently reside?
State [Drop Down]
County [Drop Down]

Q13. How do you describe your sex?
o Male/Man
o Female/Woman
o Prefer not to answer

Q14. How old are you (in years)?
o 18–24 years
o 25–34 years
o 35–44 years
o 45–54 years
o 55–64 years
o 65 years and older

Q15. For this survey, Hispanic, Latino, or Spanish origins are considered an ethnicity and not considered a race. Which race best describes you?
o American Indian or Alaska Native
o Asian
o Black or African American
o Native Hawaiian or Pacific Islander
o White
o Other
o Prefer not to answer

Q16. Are you of Hispanic, Latino, or Spanish origin?
o Yes, I am of Hispanic, Latino, or Spanish origin
o No, not of Hispanic, Latino, or Spanish origin
o Prefer not to answer

Q17. What is the highest level of education you have completed or the highest degree you have received?
o Less than high school diploma
o High school diploma or equivalent
o Post high school vocational training
o Some college, but no degree
o Associate's degree
o Bachelor's degree

o Graduate or professional degree
o Prefer not to answer

Q18. What is your annual household income?
o $49,999 or less
o $50,000 to $99,999
o $100,000 to $149,999
o $150,000 to $199,999
o $200,000 to $249,999
o $250,000 to $299,999
o $300,000 to $349,999
o $350,000 to $399,999
o $400,000 or more
o Prefer not to answer

Q19. Do you currently have health insurance coverage?
o Yes
o No
o Prefer not to answer

Q20. Generally speaking, do you usually think of yourself as a Republican, a Democrat, or an Independent?
o Strong Democrat
o Weak Democrat
o Lean Democrat
o Independent
o Lean Republican
o Weak Republican
o Strong Republican
o Prefer not to answer

Q21. Sometimes surveys can be dull, and attention can wander. To let us know that you are still paying attention, please select the baseball team that is from the City of Chicago.
o New York Yankees
o San Francisco Giants
o Texas Rangers
o Chicago Cubs
